# Episignature analysis of moderate effects and mosaics

**DOI:** 10.1038/s41431-023-01406-9

**Published:** 2023-06-26

**Authors:** Konrad Oexle, Michael Zech, Lara G. Stühn, Sandy Siegert, Theresa Brunet, Wolfgang M. Schmidt, Matias Wagner, Axel Schmidt, Hartmut Engels, Erik Tilch, Olivier Monestier, Anne Destrėe, Britta Hanker, Sylvia Boesch, Robert Jech, Riccardo Berutti, Frank Kaiser, Bernhard Haslinger, Tobias B. Haack, Barbara Garavaglia, Peter Krawitz, Juliane Winkelmann, Nazanin Mirza-Schreiber

**Affiliations:** 1 Neurogenetic Systems Analysis Group, Institute of Neurogenomics, Helmholtz Munich, 85764 Neuherberg, Germany; 2 Institute of Neurogenomics,Helmholtz Munich, 85764 Neuherberg, Germany; 3 Institute of Human Genetics, Technical University of Munich, School of Medicine, 81675 Munich, Germany; 4 Institute of Medical Genetics and Applied Genomics, University of Tuebingen, 72076 Tübingen, Germany; 5 Centre for Rare Diseases, University of Tuebingen, 72076 Tuebingen, Germany; 6 Department of Pediatric and Adolescent Medicine, Medical University of Vienna, 1090 Wien, Austria; 7 Neuromuscular Research Department, Center for Anatomy and Cell Biology, Medical University of Vienna, 1090 Vienna, Austria; 8 Institute of Human Genetics, School of Medicine, University Hospital Bonn, 53127 Bonn, Germany; 9 Centre de Génétique Humaine, Institut de Pathologie et de Génétique ASBL, 6041 Gosselies, Belgium; 10 Institute of Human Genetics, Universitätsklinikum Schleswig-Holstein, 23538 Lübeck, Germany; 11 Department of Neurology, Medizinische Universität, 6020 Insbruck, Austria; 12 Department of Neurology, Charles University, 1st Faculty of Medicine and General University Hospital in Prague, 12108 Prague, Czech Republic; 13 Institute of Human Genetics, Universitätsklinikum Essen, 45122 Essen, Germany; 14 Department of Neurology, Technical University of Munich, School of Medicine, 81675 Munich, Germany; 15 Fondazione IRCCS, Istituto Neurologico Carlo Besta, 20133 Milano, Italy; 16 Institute for Genomic Statistics and Bioinformatics, Universität Bonn, 53127 Bonn, Germany; 17 Chair of Neurogenetics, Technical University of Munich, School of Medicine, 81675 Munich, Germany; 18 Munich Cluster for Systems Neurology (SyNergy), 81377 Munich, Germany

**Keywords:** DNA methylation, Diagnostic markers, Movement disorders, Neurodevelopmental disorders, Predictive markers

## Abstract

DNA methylation classifiers (“episignatures”) help to determine the pathogenicity of variants of uncertain significance (VUS). However, their sensitivity is limited due to their training on unambiguous cases with strong-effect variants so that the classification of variants with reduced effect size or in mosaic state may fail. Moreover, episignature evaluation of mosaics as a function of their degree of mosaicism has not been developed so far. We improved episignatures with respect to three categories. Applying (i) minimum-redundancy-maximum-relevance feature selection we reduced their length by up to one order of magnitude without loss of accuracy. Performing (ii) repeated re-training of a support vector machine classifier by step-wise inclusion of cases in the training set that reached probability scores larger than 0.5, we increased the sensitivity of the episignature-classifiers by 30%. In the newly diagnosed patients we confirmed the association between DNA methylation aberration and age at onset of KMT2B-deficient dystonia. Moreover, we found evidence for allelic series, including *KMT2B*-variants with moderate effects and comparatively mild phenotypes such as late-onset focal dystonia. Retrained classifiers also can detect mosaics that previously remained below the 0.5-threshold, as we showed for *KMT2D*-associated Kabuki syndrome. Conversely, episignature-classifiers are able to revoke erroneous exome calls of mosaicism, as we demonstrated by (iii) comparing presumed mosaic cases with a distribution of artificial in silico-mosaics that represented all the possible variation in degree of mosaicism, variant read sampling and methylation analysis.

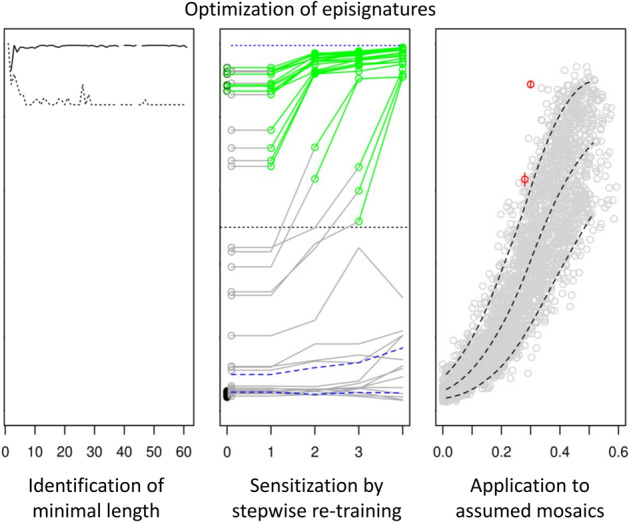

## Introduction

Disease states come with epigenetic dysregulation, especially in case of Mendelian disorders of the epigenetic machinery (MDEM) [1], which may leave specific traces in DNA methylation that can be read out as disease biomarkers. Thus, easily accessible “episignatures” have been defined for more than half of the MDEM [2, 3]. We [4] and others [5, 6] recently added histone-lysine N-methyltransferase 2B (KMT2B)-associated dystonia type 28 to that list. Moreover, we showed that disease-associated methylation aberration correlates with the age at dystonia-onset.

Episignature-classifiers are established by training a classifier such as a support vector machine (SVM) on disease-associated CpG-sites [7]. Sensitivity and specificity of the SVM-classifier depend on its training conditions: Including other MDEM in the control set, as in multiclass SVM-training [2, 7], improves the classifier’s specificity. To improve the sensitivity for intermediate cases with SVM probability scores <0.5 [8] we performed step-wise re-training of the classifier, including all newly recognized cases with scores ≥0.5 in the next training step.

Mosaicism is a challenge for the use of episignatures [2] since mosaics account for a relevant proportion of isolated disease cases. In intellectual disability, a leading phenotype also in MDEM, 6.5% of presumed de novo germline mutations have been reassigned as mosaics [9]. However, episignature-classifiers may fail to identify mosaics because of the subset of non-affected cells. On the other hand, the diagnosis of mosaicism may be erroneous due to limitations of the sequencing analysis pipeline. We used episignature-classifiers as independent tools to identify such errors.

## Materials and Methods

### Study participants and variant detection

The study comprised 268 individuals, including 35 with *KMT2B-*variants, 20 with *KMT2D-*variants, 19 training controls, and for e.g. specificity analysis, 194 independent controls with and without variants in MDEM-genes (Supplementary Table 1). Variants in *KMT2B* and *KMT2D* were detected by standardized short-read exome sequencing with read depths between 15 and 475 (mean ± SD = 199 ± 108). All participants or their guardians provided written informed consent according to the ethics research board-approved protocols of the contributing centers and all procedures were performed in accordance with the Declaration of Helsinki.

### DNA methylation analysis and quality control

Genomic DNA was extracted from peripheral blood leukocytes by standard methods. Genome-wide DNA methylation of about 850,000 CpG-sites was interrogated by Illumina MethylationEPIC BeadChip according to the manufacturer’s protocol as described previously [4]. CpG-sites with detection *p*-value > 0.01, on sex chromosomes, at known single nucleotide polymorphisms, with cross-reactivity, or call rate < 95% were excluded from downstream analyses as well as samples with mean detection *p*-value > 0.05 or call rate < 95%. After background correction and normalization using *minfi* [10], the percentage of each CpG-methylation in each individual was assessed as beta value (*β*) and expressed as *M*-value=log2(*β*/(1−*β*)). *Minfi* and all consecutive analyses were performed in R 3.6.3 software [11]. For outlier detection, we derived the 99%-confidence ellipse from the first two principal components of the 694,532 generally available CpG-sites. All study individuals were within or at the border of that ellipse (see Supplementary Material).

### EWAS, mRMR feature selection, and SVM-training

Differentially methylated CpG-sites were detected by epigenome-wide association analysis (EWAS) using *limma*_3.42.2 [12] regressing *M*-values on mutation status, sex, age, and Houseman-estimates of white blood cells. 19 controls of both sexes, 2–50 years old, matched the cases’ range of 0–51 yrs and were very unlikely genetically and phenotypically to have methylation aberrations. Cases were chosen as described below (“stepwise re-training”). Primary selection of CpG-sites from EWAS on M-values required genome-wide significance ( < 5x10E-08) and an absolute average difference >0.4.

To find an optimal selection among these sites with regard to their correlation structure we applied the bootstrap ensemble variant (mRMRe.b) of the minimum-redundancy-maximum-relevance feature selection algorithm (*mRMRe_2.1.2*) [13]. It first searches the CpG-site *x*_*1*_ that has the highest mutual information *MI* with the phenotype *y*, *MI*(*x*_*1*_, *y*)=-ln(1-*ρ*(*x*_*1*_, *y*)^2^)/2 where *ρ* is the correlation coefficient. Then, the selection *S* of CpG-sites is increased one by one, with each added site *x*_*i*_ having an optimal trade-off between maximal *MI* with *y* and minimal *MI* with the previously selected sites *x*_*j*_. This is achieved by finding the site *x*_*i*_ with maximal score *MI*(*x*_*i*_,*y*)-Σ_*j∈S*_*MI*(*x*_*i*_, *x*_*j*_)/|*S* | . Since this classical mRMR may miss the global optimum, however, ensemble versions have been developed which combine the results of *m* classical mRMR runs that are either started from each of the *m* features with the top *MI*(*x*,*y*) values or, what turned out to be even better, are run on *m* bootstraps of the examined individuals. We used *m* = 20 bootstraps of the case-control dataset, each running classical mRMR with the recommended max( | *S* | ) = 15 CpG-sites, and united all CpG-sites selected by the 20 runs. (Smaller values of *m* and max( | *S* | ) were used, however, for reducing the episignature length; see below).

On the *M*-values of the so selected CpG-sites an SVM classifier was trained (*e1071* R package) [14] with linear kernel (cost-C) and 10-fold cross-validation. Platt’s [15] probability scores with cut-off = 0.5 were used in keeping with Aref-Eshghi [7].

### Stepwise re-training of the classifier

For stepwise re-training, the initial case set consisted of patients (7 in case of *KMT2B*, 8 in case of *KMT2D*) with unambiguously pathogenic loss-of-function variants and without evidence of potential mosaicism. All tested patients whose SVM-score surpassed the level of 0.5 were then included in the case set of the first re-training step. This was repeated in further re-training steps at least until the case set did not change anymore. Specificity and quantiles (50th and 95th) were calculated in 194 individuals who were not involved in any classifier training.

### Classifier performance as function of the episignature length

For evaluation of classifier performance as function of the episignature length *k* of CpG-sites, we selected the sites as described above but increased the number *b* of bootstraps from 1 to 20 and the solution length *s*=max( | *S* | ) of the mRMR runs on each of the bootstrap from 2 to 15. This produced 20*(15–1) = 280 classifiers with 2≤ *k*< max(b*s), max(*k*) being 144 for the *KMT2B* classifiers and 68 for the *KMT2D* classifiers. For each *k*, we then averaged the specificities and pseudo-sensitivities of the classifiers of that length, with the specificity determined in 194 independent controls, and the pseudo-sensitivity being the number of variant-carriers verified as positive divided by the maximal number of such positives identified by any of the 280 classifiers.

### Evaluation of presumed mosaics

Potential mosaicism was assumed if sampling variance of sequencing reads could not sufficiently explain the difference between the observed and the expected variant read count *n*/2 under the assumption of non-mosaicism, i.e., if the absolute of that difference was larger than 2 standard deviations (SD) of a binomial distribution Bin(*n*,*p*), that is, >2(*np*(1-*p*))^½^=√*n*, with read depth *n* and *p* = 0.5.

All potential mosaics were included in the analysis under the re-training paradigm described above. For discovering erroneous mosaics by comparison with in silico-mosaics, the classifiers were used without re-training, however. Moreover, two cases were left out from the training set and used for construction of in silico-mosaics (see below) in order to have sufficiently many cases to choose from. All variant-carriers identified by the classifier and without evidence of potential mosaicism were also included in the set from which the affected parts of the in silico-mosaics was chosen randomly. The unaffected counterparts were selected randomly from a set of 67 individuals, aged 2–78 yrs (mean ± SD = 20 ± 18), lacking any evidence of MDEM, and not used in classifier training. The degree *d* of mosaicism ( = proportion of the affected part) was selected randomly from a uniform distribution *U*(0,1), and the DNA methylation beta values of the mosaic were then assumed to be the proportionally weighted averages of the two parts, *β*(in silico-mosaic)=d*β(affected)+(1-*d*)*β(unaffected), from which the *M*-values were calculated as indicated above. Each in silico-mosaic was assigned with a sequencing read depth *n*, randomly selected from a normal distribution with mean ± SD and lower-end truncation as observed for the gene-specific read depths of the cases with variants in the respective gene (184 ± 93 and > 47 for *KMT2B*, 213 ± 121 and > 14 for *KMT2D*). Then, to model sampling variance of the sequencing reads, the variant read count of the in silico-mosaic was selected randomly from a binomial distribution Bin(*n*,*p*) with *p* = *d*/2, considering heterozygosity of the causative variants in *KMT2B-* and *KMT2D*-related disorders. With multiple different ways of leaving out 2 cases from the training set (7!/2!/5! = 21 for *KMT2B* and 8!/2!/5! = 28 for *KMT2D*), we created 150 in silico-mosaics for each possibility which, together with the potential mosaic cases under examination, then received SVM-scores by the respective classifier. Thus, we generated 21*150 = 3150 in silico-*KMT2B*-mosaics and 28*150 = 4200 in silico-*KMT2D*-mosaics with read depths, variant read counts, and SVM-scores. For each gene, 3000 randomly selected in silico-mosaics were then sorted according to their relative variant read counts in order to calculate their 5th and 95th SVM-score quantiles in 0.025-sized bins of relative variant read count between 0 and 0.5, followed by smoothing using the default *loess*-algorithm in R [11]. From the 21 respectively 28 SVM-scores derived for each potential mosaic under examination, individual means and 95%-confidence intervals were calculated. (See Supplementary Material for a flow chart of the evaluation of presumed mosaics.)

### Mean normalized methylation deviation versus age at dystonia-onset

As described previously [4], the individual mean normalized methylation deviation was the average of the absolute *z*-values at the CpG-sites of the (not re-trained) episignature. The *z*-value of a CpG-site was the difference of the individual’s *M*-value from the mean of the *M*-values at that site in controls, divided by their SD in controls. The association with the age at dystonia-onset was assessed by Cox-proportional-hazards-regression with right-censoring (if dystonia had not yet occurred at the time of examination) and maximum-likelihood-ratio significance testing (R *survival* package) [16].

## Results

### Optimizing episignatures by mRMRe.b

**Fig. 1:**
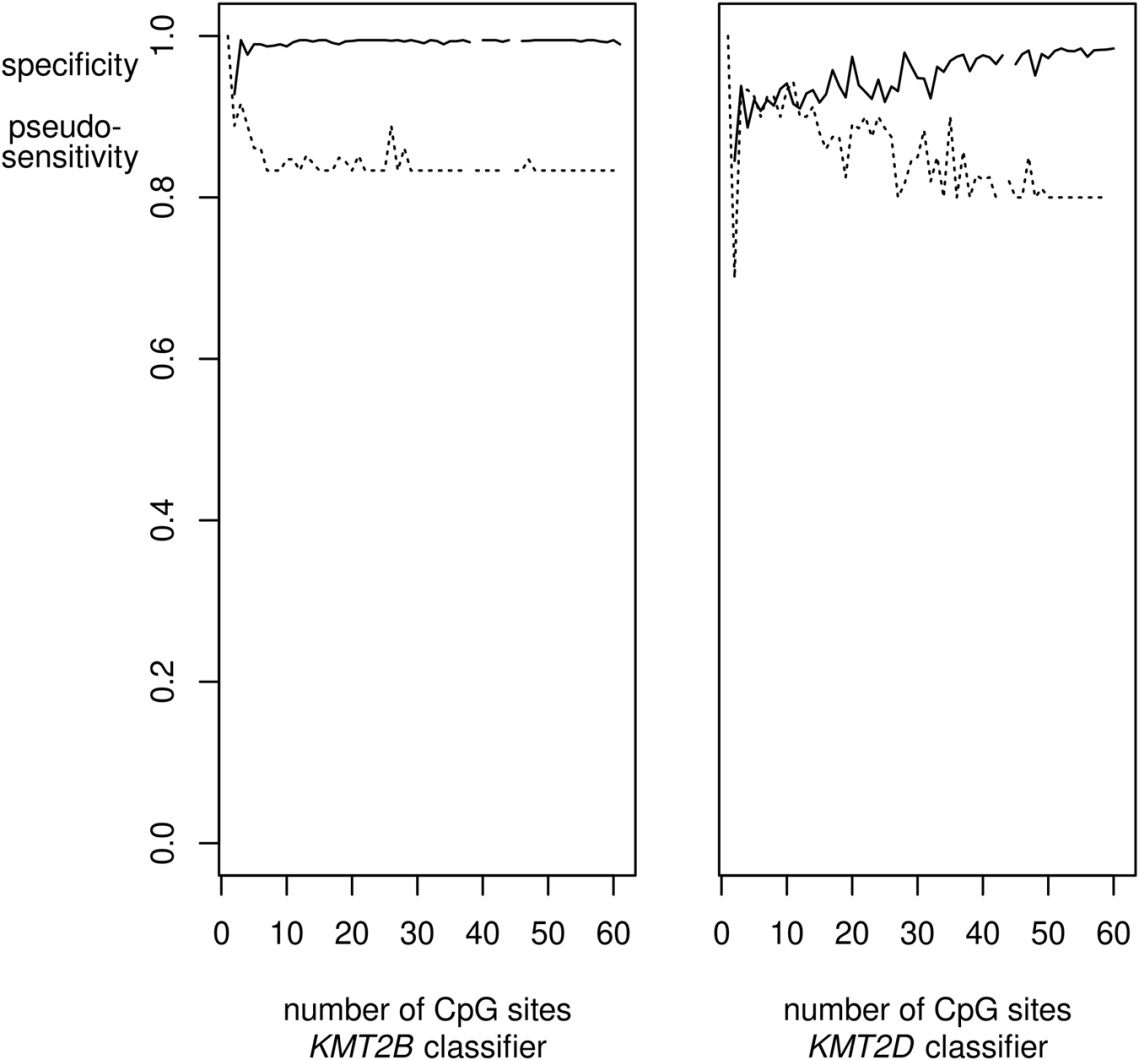
SVM-classifier performance as a function of episignature length. The performances of SVM episignature-classifiers for *KMT2B* and *KMT2D* after the third re-training (cf. Fig. 2) are shown as a function of their length, that is, the number *k* of CpG-sites included in the episignature. Selection of sites by the ensemble bootstrap version of the mRMR algorithm was varied by varying the number of bootstraps and the length of the mRMR solutions. For each *k* the average specificity of the classifiers of that length was calculated (solid line). Analogously, a pseudo-sensitivity (dashed line) was calculated as the average number of variants verified as pathogenic by the classifiers of length *k* divided by the maximal number of (seemingly) verified variants by any of the classifiers for the respective gene. The specificities reached their plateaus for *k* ≥ 4 in case of *KMT2B* and *k* ≥ 49 in case of *KMT2D*. Pseudo-sensitivities stabilized at *k* ≥ 30 and *k* ≥ 49, respectively. Since not all *k* were realized by the selection procedure, the curves have small gaps.

EWAS results may qualify a large number of CpG-sites for inclusion in an episignature-classifier (we identified >1000 in the *KMT2B* EWAS). These sites are likely correlated, that is, redundant when used in classifiers or predictors. Therefore, we selected the sites for a classifier by applying the mRMRe.b-algorithm [13, 17] to the initial EWAS-based selection. Performing 20 bootstraps with solution length max( | *S* | ) = 15 each, we reduced the number of sites for the classifier by one order of magnitude to 144 in case of *KMT2B* and by half to 68 in case of *KMT2D* in the third re-retraining (Supplementary Table 2). These classifier sizes still had a considerable safety margin. In fact, by varying max( | *S* | ) between 2 and 15 and the number *m* of bootstraps between 1 and 20, we found that in terms of specificity and sensitivity, classifiers reached stable performance ( ≤ 1.5% deviation from window mean) already with 4 and 30 CpG-sites, respectively, in case of *KMT2B*, and with 49 sites for both specificity and sensitivity in case of *KMT2D* (Fig. 1).

### Optimizing episignatures by stepwise re-training

**Fig. 2:**
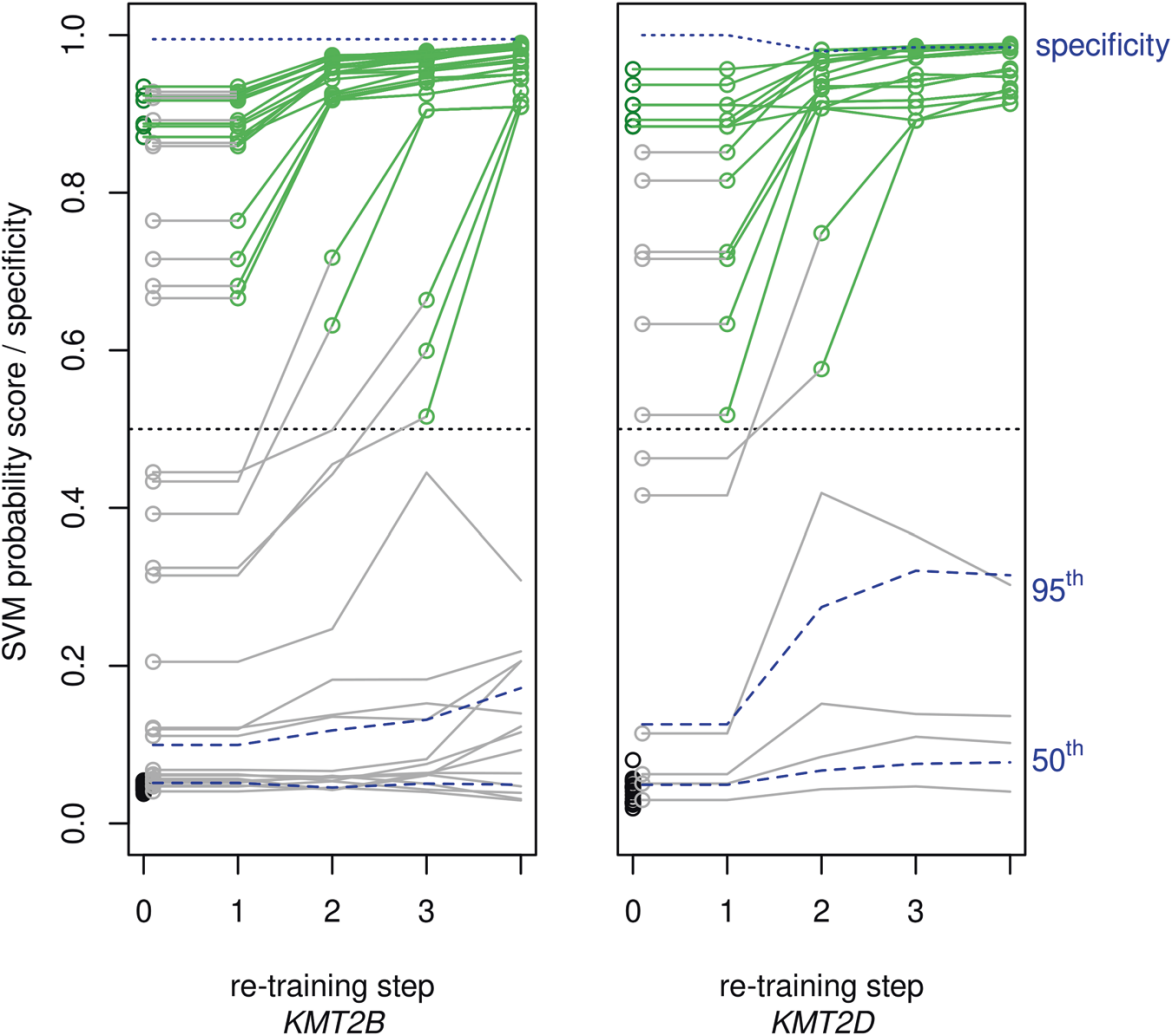
SVM probability scores with stepwise re-training of classifiers. SVM-classifier training started with cases (dark green) with obviously pathogenic, that is, non-mosaic loss-of-function variants (step 0). This initial classifier was applied to cases with variants (gray) of various levels of pathogenic significance. The cases in whom this produced an SVM probability score > 0.5 were then included in the case set (light green) for re-training of the classifier. Novel cases to be thus included in re-training were detectable up to the 3rd re-training of the *KMT2B* classifier and up to the 2nd re-training of the *KMT2D* classifier. The 19 controls (black) were the same in all training steps. Dashed blue lines indicate 50th ( = median) and 95th quantiles of the classifiers’ SVM probability scores in a set of 194 independent samples with and without variants in genes of the epigenetic machinery other than the gene under examination. The upper dotted line indicates the classifiers’ specificities as determined in these independent control samples.

Stepwise re-training of the classifier by inclusion of diagnosed cases in the training set increased the sensitivity of the classifiers, essentially without loss in specificity (Fig. 2). Beyond the mere increase of the number of cases and the concomitant balancing of the case-control proportion, the re-trained classifier profited from widening the spectrum of effect sizes in the training set. Initial classifier training comprised only cases with obviously pathogenic variants, that is, loss-of-function variants without evidence of mosaicism. The initial classifier was then applied to all samples, including those that carried a variant in the respective gene and were received too late or had unclear pathogenicity attributed to the variant (e.g., missense-VUS; see Supplementary Tables 3, 4). Thereby, 10 pathogenic *KMT2B-*variants and 6 pathogenic *KMT2D*-variants were verified as pathogenic and included in the case set for the first re-training of the respective classifier. Cases to be included in that way came up until the 3rd re-training of the *KMT2B*-classifier and the 2nd re-training of the *KMT2D*-classifier. Maximal re-training of the classifiers identified 7 more individuals as carrying pathogenic variants, including 3 *KMT2B-*variants (one of them being present in 3 related individuals) and 2 *KMT2D-*variants, thus increasing the classifiers’ sensitivities by 3/10 = 30% for *KMT2B-*variants and 2/6 = 33% for *KMT2D-*variants, respectively, while the specificities as determined in a set of 194 independent controls remained close to 1 (0.99 and 0.985, respectively; see Fig. 2 and Supplementary Table 1). The two *KMT2D-*variants verified only after re-training were de novo stop mutations, occurring as mosaics with low variant read count proportions (0.19 and 0.23) and strong deviation from the expected heterozygous read count (by 9.8 and 6.3 binomial SD, respectively). None of the cases whose scores remained <0.5 even after re-training were mosaics.

Even the maximally re-trained classifier may have missed some pathogenic variants. 4 dystonia cases with VUS in *KMT2B* ranged above the 95th percentile of the independent controls after the third re-training but failed to reach SVM-scores ≥0.5 (Fig. 2). Phenotypes in these 4 cases were less severe than in classical *KMT2B*-related dystonia, including generalized dystonia with no neurodevelopmental comorbidity (2/4), minor neurodevelopmental disturbances without dystonia (1/4), and adult-onset isolated focal (cervical) dystonia (1/4). Remarkably, transmission to seemingly unaffected offspring was seen in this variant category. Moreover, when comparing the SVM-scores of VUS-carriers below the 95% percentile with those of independent controls below that level by Wilcoxon rank sum test (one-sided with continuity correction; individuals from the same family being represented by only one value, i.e., their mean), the VUS-carriers ranged significantly higher (*p* = 0.03 for *KMT2B* and *p* = 0.02 for *KMT2D*). *KMT2B* and *KMT2D-*variants with re-trained SVM-scores above the 95th percentile of the independent controls are listed in Supplementary Tables 3, 4, respectively.

### DNA methylation deviation versus age at onset of KMT2B-deficient dystonia

Among the individuals with *KMT2B-*variants that had not been examined previously already [4] which implies that they also were not included in the initial training of the present study, 8 patients with available data on age at dystonia-onset were verified to be KMT2B-deficient (directly or after re-training of the classifier). In these patients we examined the correlation between the age at dystonia-onset and the average normalized deviation of *M*-values of the CpG-sites contained in the initial classifier as described previously [4]. We replicated the then described negative correlation with a significance of *p* = 0.017 (maximum-likelihood-ratio test after Cox-proportional-hazards regression with right-censoring in 2 cases in whom dystonia had not yet occurred at the time of examination). Similar to the previous analysis, the average of the normalized *M*-values was 2.9 and 3.7 in individuals with no onset before adulthood and 7.2 for onset at preschool age. Using age as proxy of age at onset in the 2 censored cases, Pearson correlation was −0.55, corresponding to *r*^2^ = 30% of the variance in age at onset explained by the average normalized methylation deviation. This is lower than the previously observed *r*^2^ = 57% [4], possibly due to a winner’s curse in the previous analysis. Testing of the age at onset against the SVM-scores of the primary classifier (instead of the average normalized methylation deviation) gave less significant results (*p* = 0.038, *r*^2^ = 0.24) but the difference of the correlation coefficients was not significant (*p* = 0.31, William’s test).

### Evaluation of presumed mosaics

**Fig. 3:**
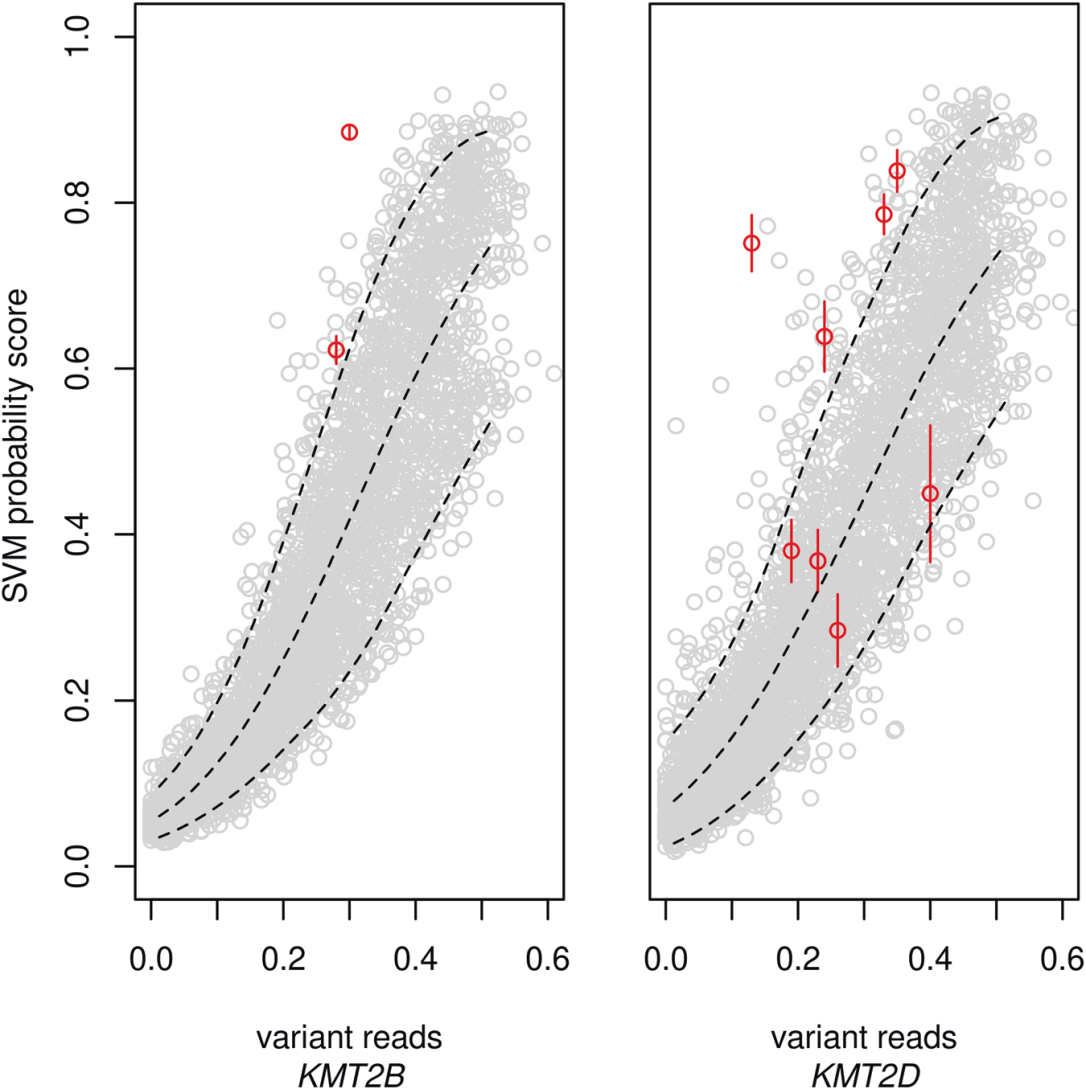
Analysis of presumed mosaics. For suspected mosaics (red), that is, cases with variant read calls diverging by more than 2 binomial SD from expectation (i.e., from half of the total read number), the SVM probability scores and variants’ read proportions were compared to those of in silico-synthesized mosaics (grey) which represented the potential variations due to degree of mosaicism, read depth, variant read sampling, methylation analysis, and classifier training. Dashed lines indicate the 5th and 95th percentiles of the SVM-scores in the synthesized mosaics. The variation in classifier training resulted in variation of the SVM-scores of the suspected mosaics (vertical red lines indicating the 95%CI). Note that the classifiers were varied by running through all possibilities of leaving out 2 cases from the training set, reducing their power as compared to the classifiers in Fig. 2 that were trained on the complete sets. The suspected *KMT2B* and *KMT2D* mosaics above the 95th percentile were reassessed (Table 1) and at least 4 of them were found to be erroneous outputs of the automated exome analysis pipeline due to poor read depth or insufficient assignment of indel reads.

We examined cases with strongly diverging variant read calls as potential mosaics, that is, all cases with variant read call numbers diverging from the expected value (=*n*/2) by more than 2 SD ( = 2(*n*/4)^1/2^ = √*n*) of the theoretical binomial sampling distribution Bin(*n*,*p*) in non-mosaic cases with read depth *n* and sampling probability *p* = 0.5. Since *n* differed from case to case (mean ± SD = 184 ± 93 for *KMT2B*-variants and 213 ± 121 for *KMT2D*-variants), the threshold expressed as variant read call proportion also depended on the individual *n* according to (n/2-√*n*)/n = 0.5-1/√*n*. The variant read call proportions of the potential mosaics that we examined thus varied between 0.13 and 0.4 (Fig. 3). They comprised 2 cases with *KMT2B*-variants and 8 with *KMT2D-*variants, 5 being indels and 5 single nucleotide variants (SNV), all of them causing loss-of-function with the possible exception of one in-frame duplication (see Supplementary Tables 3 and 4).

We compared the SVM-scores of these potential mosaics with the score distribution of artificial, in silico-synthesized mosaics. These artificial mosaics accounted for all possible sources of variation, that is, variable degree *d* of mosaicism, variable, i.e. randomly selected empirical cases and non-cases representing the methylations in the mutated and non-mutated parts of the mosaic, variable sequencing read depth *n*, and variable sampling deviation of reads. Moreover, we also varied the sets of cases used for SVM-training and cases used for composing the in silico-mosaics. Expectedly, the overall distribution of the artificial mosaics was rather broad (Fig. 3). Nonetheless, the analysis highlighted 6 suspected mosaics as possibly erroneous as their SVM probability scores were above the 95th percentile of the artificial mosaics. The 6 variants included 1 SNV and all 5 indels, reminding of the fact that indel detection by exome sequencing is insufficient [18]. When these 6 cases were re-examined, the initial assumption of mosaicism was found to be erroneous in both *KMT2B* mosaics and in 2 of the *KMT2D* mosaics, while 2 other *KMT2D*-indels still remained possible mosaics after re-examination (Table 1): Direct inspection by Integrative Genomics Viewer (IGV) in case of the tandem duplication c.6245_6266dup in *KMT2B* additionally revealed 2 reads where the duplication was misinterpreted as mismatches and 49 non-informative reads which started or ended within the duplicated region and may well have been derived from the duplicated allele. Assuming an unbiased distribution of allelic reads, the initial variant read count by the automated exome pipeline (52 out of 171, resulting in a seeming allele frequency of 0.30) was thus corrected to 52 + 2 + 49/2 = 78.5, resulting in an allele frequency of 0.46 and a deviation of only 1.07 binomial SD. In case of the *KMT2B*-deletion c.3335-9_3363del, the re-analysis of reads by *DeepVariant*, a deep convolutional neural network which outperformed state-of-the-art tools [19], indicated 68 variant reads in 166 reads, strongly increasing the seeming variant allele frequency from 36/129 = 0.28 to 0.41 which corresponded to a borderline deviation of 2.3 binomial SD. SVM probability scores of 4 *KMT2D* mosaic candidates were above the 95th percentile of the artificial mosaics. One of them showed the previously described pathogenic splice site variant c.510 G > A [20] in only 2 of 15 reads. Despite a deviation from the expected variant read count by 2.9 binomial SD, this case was very unlikely to be a mosaic because the variant was inherited from the mother. The other 3 cases carried indels. At least one of them was very unlikely to be a mosaic upon *DeepVariant* examination which elevated the relative variant allele count above 50% (Table 1).

**Table 1:** Re-examination of suspected mosaics with SVM-scores above the 95th percentile of synthetic mosaics.

Gene	DNA variant	Type	Primary exome data			Method of re-examination	Data after re-examination			Evaluation of mosaicism and variant allele freq (VAF) after re-examination
			Read depth	Variant reads	Deviation [binom. SD]		Read depth	Variant reads	Deviation [binom. SD]	
*KMT2B*	c.3335-9_3363del	indel	129	28%	−5.00	Direct inspec-tion of IGV	166	41%	−2.34	Unlikely. VAF now close to 50%.
*KMT2B*	c.6245_6266dup	indel	171	30%	−5.12	DeepVariant	171	46%	−1.07	Unlikely. VAF now close to 50%.
*KMT2D*	c.2091dup	indel	51	35%	−2.14	DeepVariant	60	32%	−2.84	Possible. VAF deviation further increased.
*KMT2D*	c.510G>A	SNV	15	13%	−2.87	Pedigree analysis	15	13%	−2.87	Unlikely. Variant from mother.
*KMT2D*	c.8443dup	indel	370	24%	−10.00	DeepVariant	211	25%	−7.23	Possible. VAF deviation still very large.
*KMT2D*	c.15163_15168dup	indel	252	33%	−5.40	DeepVariant	216	57%	2.04	Unlikely. VAF larger than 50%.

## Discussion

We optimized episignature-classifiers and their application with respect to several aspects. First, we reduced the size of the classifiers which usually comprise 100 to 500 CpG-sites [2] by removing redundancy without loss of accuracy. We did so by applying the bootstrap ensemble version of the minimum-redundancy-maximum-relevance feature selection algorithm (mRMR) [13, 17] whose name explains its basic idea. This size reduction was most efficient in case of KMT2B-deficiency where the set of CpG-sites with epigenome-wide significance could be reduced by almost 2 orders of magnitude to minimally 30 without detectable loss in the classifier’s accuracy (Fig. 1). In case of KMT2D-deficiency the maximal reduction was less pronounced, i.e. by about half to minimally 49 CpG-sites. The potential for reduction differs between diseases because the specific deficiencies do not have the same effect (and effect size) on DNA methylation. Others previously reduced classifier redundancies by removing CpG-sites with correlation > 80% [7] or ≥ 90% [21, 22]. However, the accepted level of remaining redundancy appeared to be arbitrary in these studies and the selection of individual CpG-sites was not a direct function of the trade-off between redundancy and relevance. Moreover, because their further correlation patterns may differ, it is not irrelevant which of two correlated CpG-sites is selected. The mRMR-algorithm provides a plausible selection rationale. Nonetheless, it may still miss the globally optimal classifier. In its bootstrap ensemble version, which we applied, the optimization is further improved without exceeding computation time [13]. (Note added in proof: During preparation of this manuscript Zhang et al. [23] reported on feature selection by mRMR when relating fetal intolerance of labor to maternal blood cell DNA methylation.).

Second, we optimized the sensitivity of the episignature-classifier by repetitively re-training the classifier with recursive inclusion of newly diagnosed cases into the classifier’s training set. Each episignature-classifier of a monogenic disorder necessarily must first be trained on undoubtedly positive cases. “Undoubtedly” implies a trend towards loss-of-function variants in the causative gene which usually have strong effects, however. This limits the sensitivity of classifiers in case of disorders in which the phenotypic severity relates to the remaining genetic effect. That limitation was substantially reduced by stepwise re-training of our *KMT2D* and *KMT2B* classifiers, increasing their sensitivity by 30% while their high specificity was preserved. Sooner or later, such re-training of classifiers runs into contingent and necessary restrictions, however. Contingently, the recursive re-training may break off if no case of intermediate severity comes up anymore to be included in the next training step. Thus, at least 4 of our dystonia patients with VUS in *KMT2B* whose SVM probability scores remained below 0.5 even after maximal re-training, likely are KMT2B-deficient because the scores were still above the 95th percentile of independent controls. A necessary restriction of the re-training method beyond inclusion of moderate-effect variants is given by the unavoidable trade-off between sensitivity and specificity if there is a continuous allelic series of effect strengths. Including variants of lower and lower effects in the training set will, at some point, impair the specificity of the classifier due to random variation in the control set or residual cross-sensitivity of the classifier for other disorders. The latter problem can be remedied by including samples of the respective disorders in the control set of the SVM-training or even of the EWAS [2]. We did not do so since we wanted to see any impact of the re-training on the specificity. As indicated above, this impact was very small and the specificities remained close to 1 ( ≥ 0.98). Expectedly, a few cases of disorders with related episignatures [3, 24] came up such as a DNMT1-deficient sample in case of the *KMT2B* classifier and samples from BAFopathies or Kabuki type 2 in case of the *KMT2D* classifier.

We previously found evidence of allelic series in *KMT2B*-associated dystonia where the age of dystonia-onset was associated with the degree of KMT2B-deficient methylation deviation [4]. Other made analogous observations in other disorders such as the Au-Kline syndrome [8], for instance, where intermediate severities correlated with intermediate episignature-classifier scores. We now confirmed this association in independent samples of KMT2B-deficient dystonia. Analogous association between phenotypes and methylation deviation may be detectable for reliably quantifiable phenotypes in other MDEM.

Cases above the 95th percentile in independent controls but below the 0.5-probability score of the maximally re-trained classifier also had no or late-onset (mean = 26 years) dystonia. These four cases included an adult patient with isolated focal cervical dystonia as the only symptom, highlighting the possibility that moderate KMT2B-deficiency may play a causative role in a set of dystonia patients much larger than previously thought. Even the *KMT2B* VUS cases below the 95th percentile of the probability score ranked significantly higher than the independent controls in that range. The same was true for *KMT2D* VUS. These findings raise the possibility that those deficiencies contribute with small effect to polygenic forms of the respective disorders, that is, dystonia and intellectual disability, respectively. However, this would imply a low pathogenicity-threshold of the histone and DNA methylation deviations.

The optimal trade-off between sensitivity and specificity of a tunable biomarker such as a classifier score varies depending on the biomarker’s concrete use and on the prior diagnostic probability. For instance, if the biomarker is the only available parameter to diagnose a monogenic disorder, its specificity should not be much compromised. On the other hand, if the classifier is used to exclude the pathogenicity of a VUS, high sensitivity is desirable. As shown in Fig. 2, there are VUS in *KMT2B* and *KMT2D* whose classifier scores remained close to the median of independent controls even after maximal re-training.

Sensitivity is also crucial when episignature-classifiers are applied to mosaics since only a fraction of the examined blood cells display the deviation in methylation. Indeed, two of eight potential *KMT2D* mosaics were only detected when the classifier had become more sensitive after re-training (Fig. 2). Their variants’ read proportions were as low as 0.19 and 0.23. Montano et al. [25] recently described a *KMT2D* mosaic with variant read proportion of 0.11 whose classifier score of 0.2 clearly ranged above the scores of controls but failed to reach the level of 0.5.

Regular diagnostic use of episignatures for evaluation of mosaic states has recently been called for, as the latter may account for a relevant proportion of disease cases [2, 9]. Collecting sufficiently many mosaic samples for each degree of mosaicism in order to train the respective classifiers will be difficult, however. Therefore, as the third optimization developed by this study, we simulated mosaic states in silico by pairwise combination of methylation data from sets of non-mosaic cases and of non-affected assuming that the specific DNA methylation aberration in blood of an MDEM mosaic is displayed only by cells that carry the defect of the epigenetic machinery. Besides the variation in methylation assessment which was represented by the empirical data sets used for their construction, the distribution of the in silico-mosaics also realistically represented the variation of the degree of mosaicism and the variation of the proportion of variant sequencing reads. When we compared the 10 potential mosaic cases in our cohorts with the distribution of these in silico-mosaics (Fig. 3), we identified 6 outliers of whom 5 carried indel variants which are notoriously difficult to adequately detect by exome sequencing [18]. Re-evaluating the sequencing and family data of the 6 outliers, mosaicism turned out to be unlikely in at least 4 of them. These findings benchmarked the usefulness of the in silico-mosaics. Interestingly, after re-evaluation of the outliers, none of the KMT2B-deficient patients appeared to be an obvious mosaic. The difference to KMT2D-deficient patients - of whom at least 4 SNV-carriers complied with mosaicism according to pedigree, exome, and episignature data - was striking, as our study included more cases with KMT2B-deficiency than cases with KMT2D-deficiency. Indeed, *KMT2B* mosaics have not yet been published either, as opposed to 14 reported *KMT2D* mosaics [20, 25, 26]. As a possible explanation of this difference, there may be interneuronal redundancy in suppression of KMT2B-deficient dystonia so that mosaics rarely develop dystonia.

## Supplementary information

Supplementary Material

Supplementary Table 1

Supplementary Table 2

Supplementary Table 3

Supplementary Table 4

## Data Availability

CpG-sites episignatures for *KMT2B* and *KMT2D* after maximal re-training are provided in Supplementary Table 2. Details of the variants in *KMT2B* or *KMT2D* with evidence of pathogenicity in (re-trained) episignature analyses are provided in Supplementary Tables 3 and 4.
