# Supplementary material for: Episignature analysis of moderate effects and mosaics
Source: Eur J Hum Genet. 2023 Jun 26;31(9):1032–9. doi: 10.1038/s41431-023-01406-9 (PMC10474287; doi:10.1038/s41431-023-01406-9)

**Supplementary Material**

**for**

**“Episignature analysis of moderate effects and mosaics” (Oexle et al., 2023)**

Content

1) Outlier analysis

2) Heatmaps of the maximally re-trained episignatures

3) Flow chart of the analysis of presumed mosaics using synthesized mosaics

1) Outlier analysis by principal component analysis

Plot of the first two principal components for all 268 individuals in the study based on the *M*-values of all CpG sites present after quality control. All individuals were within or at the border of a 99%-confidence ellipse (blue) of a two-dimensional normal distribution with the standard deviations of the first two principal components.

2) Heatmaps of the maximally re-trained episignatures. Each separated two major clusters, one containing all training cases and verified cases and the other all training controls.

Heatmap with hierarchical clustering of *M*-values of the 144 CpG sites in the *KMT2B*-episignature after 3^rd^ re-training as assessed in the intial training set (“KMT2B_1st_t”), all other verified carriers of causative *KMT2B*-variants (“KMT2B”), and the controls.

Heatmap of M-values of the 68 CpG sites in the *KMT2D*-episignature after 3rd re-training as assessed in the intial training set (“KMT2D_1st_t”), all other verified carriers of causative *KMT2D*-variants (“KMT2D”), and the controls.

3) Flow chart of the analysis using synthesized mosaics, exemplified for the *KMT2B* mosaics


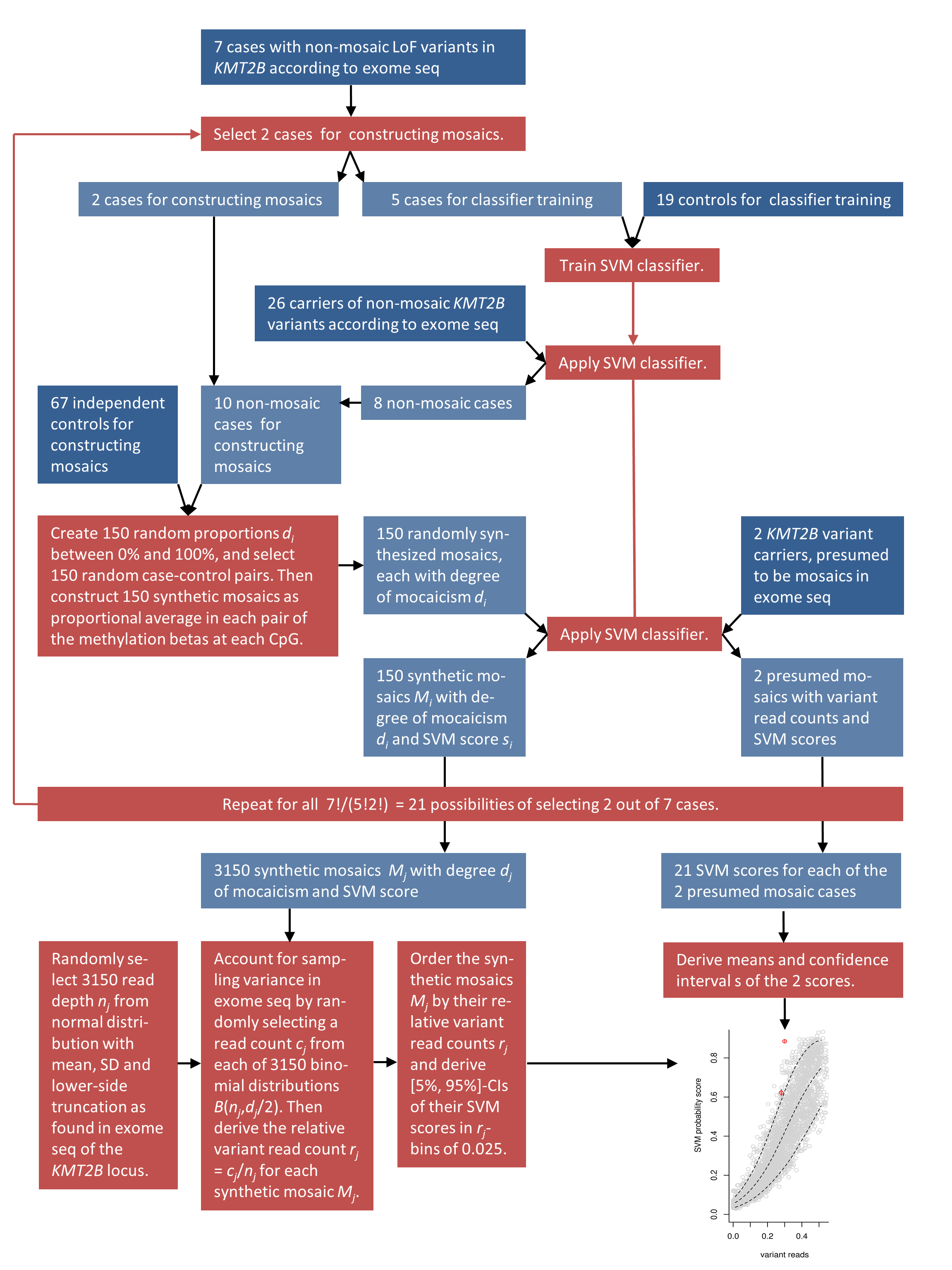

Supplement: Supplementary file 1 — Supplementary Material [file 41431_2023_1406_MOESM1_ESM.docx]
